# Clustering patterns of human papillomavirus infections among HIV-positive women in Kenya

**DOI:** 10.1186/1750-9378-8-50

**Published:** 2013-12-19

**Authors:** Salvatore Vaccarella, Hugo De Vuyst, Nelly R Mugo, Samah R Sakr, Martyn Plummer, Daniëlle A M Heideman, Silvia Franceschi, Michael Chung

**Affiliations:** 1 International Agency for Research on Cancer, 150 cours Albert Thomas, Lyon cedex 08 69372, France; 2 Department of Obstetrics and Gynecology, Kenyatta National Hospital, P.O. Box 19676, off Ngong Road, Nairobi, Kenya; 3 Coptic Hospital, P.O. Box 21570, Ngong Road, Nairobi, Kenya; 4 Department of Pathology, VU University Medical Center (VUMC), Amsterdam, The Netherlands; 5 Department of Global Health, University of Washington, 325 Ninth Avenue, Box 359909, Seattle, WA 98104, USA; 6 Department of Medicine, University of Washington, 325 Ninth Avenue, Box 359909, Seattle, WA 98104, USA; 7 Department of Epidemiology, University of Washington, 325 Ninth Avenue, Box 359909, Seattle, WA 98104, USA

**Keywords:** Human papillomavirus, HIV, Prevalence, Women, Multiple infections

## Abstract

**Background:**

HIV-positive women are at increased risk of human papillomavirus (HPV) infection, and, especially, multiple infections compared to HIV-negative women. Whether certain HPV types have a tendency to cluster in multiple infections beyond or below what would be expected by shared risk factors (e.g., sexual behavior and the degree of immunosuppression) is unclear. We, therefore, investigated clustering patterns of 44 HPV types in HIV-positive women from Kenya.

**Findings:**

HPV status was assessed on cervical scrapings from 498 women using GP5+/6+ PCR and reverse line blot. Logistic regression was used to model type-specific HPV positivity, adjusted for age, specific HPV type prevalence, CD4, combination antiretroviral therapy, and, in the Full Model, individual-level random effects that represent unobservable risk factors common to all HPV types. We found a modest excess of women with co-infections with 2 HPV types (1.12; 95% credible intervals: 1.03-1.21) in the Full Model but no significant associations of individual types. No significant deviations of observed/expected counts were observed for any 2-way combination of HPV types at either the chosen level of significance, p = 0.00005, or at p = 0.01. Findings were substantially similar when women with CIN2/3 were excluded and when they were stratified by use of anti-retroviral therapy or CD4 count.

**Conclusions:**

HPV co-infections occurred at random in the cervix of HIV-positive women as previously found in HIV-negative women. The removal of HPV types through vaccination should not result, therefore, in an increase or decrease in the prevalence of HPV types not targeted by vaccination in immunosuppressed women.

## Findings

### Introduction

HIV-positive women are at increased risk of infections with human papillomavirus (HPV) compared to HIV-negative women [1,2]. Although multiple HPV infections are particularly common in HIV-positive women, it is not clear whether certain HPV types have the tendency to cluster in multiple infections beyond or below what would be expected by shared risk factors, e.g., sexual behavior and the degree of immunosuppression. This could be relevant for the evaluation of the effects of HPV prophylactic vaccines. The removal of certain HPV types through vaccination could, in theory, indirectly increase or decrease the prevalence of other untargeted types.

Previous studies among HIV-negative women [3-7] and men [7,8] have reported a general tendency of HPV types to cluster in multiple infections. However, there has been no evidence for specific HPV types to be found together more or less often than expected by chance with other types. Significant excesses of a few HPV type combinations have been observed, but they have been demonstrated to derive from diagnostic artefacts, e.g., cross-hybridization of closely homologous HPV types using enzyme immunoassay for genotyping [8] or technical limitations of the HPV detection methods used, e.g., indirect measure of HPV52 by using a mixed probe using Roche Linear Array [3].

To date, the clustering patterns of multiple HPV infections in HIV-positive women have been evaluated in only one study based in the United States [9]. Chaturvedi et al. [9], however, only assessed clustering at the phylogenetic clade level, not individual HPV type level. We, therefore, have investigated this issue in a cross-sectional study of HIV-positive women in Nairobi, Kenya.

## Materials and methods

The study in Kenya has been described in previous publications [10-12]. Briefly, in 2009, 500 HIV-positive women, aged 18–55 years, were enrolled with the primary aim of comparing cervical cancer screening methods. After obtaining a written informed consent, cervical exfoliated cells were successfully collected from 498 women to perform HPV testing, and HPV DNA was assessed using a general GP5+/6+ primer–mediated PCR to detect 44 HPV types with reverse line blot analysis for genotyping [13]. A venous blood sample was taken to measure CD4 count. Type-specific prevalence was described in detail [10]. HPV prevalence is slightly different in the present report [10], because six women who were HPV-positive only according to HPV generic mix probes were excluded. The study protocol was approved by the Ethical Review Committees of the Kenyatta National Hospital, Kenya; the University of Washington, USA; and the International Agency for Research on Cancer, France.

Statistical methods used in the present report have been described in previous publications [3,5-8,14,15]. Briefly, a multivariate logistic regression was used to model type-specific HPV positivity. None of the sexual behavior variables collected by the enrollment questionnaire was included in the models because they were not found to be significantly associated with HPV-positivity in a previous analysis [10].

Three models were used. The Basic model included age and specific HPV type prevalence only as covariates. The Adjusted model added CD4 count (as a continuous variable), use of combination antiretroviral therapy (cART) (never, <2 years, ≥2 years) and an interaction term between CD4 counts and cART use. This model adjusted for the fact that low CD4 counts were found to be associated with higher HPV positivity and that this association was weaker among women with a prolonged cART use [10]. The Full model added individual-level random effects. Random effects quantify and account for the fact that women with several concurrent HPV types may have a higher level of risk for HPV infection compared to women with few or no HPV types, and that type-specific HPV measurements in the same woman are correlated with each other. Individual-level random effects represent unobservable risk factors common to all HPV types [3].

A Bayesian approach with Markov Chain Monte Carlo simulation was used; estimates were reported as posterior means and 95% credible intervals (95% CI). Discrepancies between the data (observed counts of co-infections per subject) and the model (expected counts of co-infections per subject) were assessed by posterior predictive two-sided p-values and measured by an observed-to-expected (O/E) ratio for each HPV co-infection. Since all possible two-way interactions between the 44 HPV types were included, this generated 946 (44 × 43/2) statistical comparisons. To minimize errors due to multiple comparisons, the Bonferroni correction was used to set the p-values thresholds in order to assess statistical significance. With 946 multiple comparisons, the Bonferroni corrected p-value threshold for type-type associations was 0.05/946 = 0.00005.

## Results

Overall HPV positivity in the 498 HIV-positive women was 67.5% (N = 336). Multiple infections were found in 56.8% (N = 191) of HPV-positive women (Table 1). According to the Basic model, there was an excess of HPV-negative women [O/E, ratio = 1.32; 95% Credible Interval (CI): 1.20-1.46]. O/E ratios for infection with 2 and ≥3 HPV types were 0.85 (95% CI: 0.81-0.89) and 1.13 (95% CI: 0.98-1.29), respectively. With the Adjusted model, the O/E ratio was 1.23 (95% CI: 1.11-1.35) for 0 HPV types, 0.90 (95% CI: 0.86-0.96) for 2 HPV types, and 1.07 (95% CI: 0.93-1.22) for ≥3 HPV types. Inclusion of CD4 counts, cART use and their interaction in the model slightly reduced the discrepancy between observed and expected counts. When the Full model was used, no substantial difference was found between observed and expected counts, with the exception of a small but significant excess of double HPV infections (O/E ratio = 1.12, 95% CI: 1.03-1.21).

**Table 1:** **Observed** (**O**) **to Expected** (**E**) **ratio of multiple infections with 44 HPV types**, **according to various models**

			**Basic model**		**Adjusted model**		**Full model**	
**No of HPV types**	**O**	**%**	**E**^**1**^	**O/E (95% CI)**^**1**^	**E**^**2**^	**O/E (95% CI)**^**2**^	**E**^**3**^	**O/E (95% CI)**^**3**^
0	162	32.5	122.8	1.32 (1.20-1.46)	131.5	1.23 (1.11-1.35)	163.1	0.99 (0.90-1.09)
1	145	29.1	175.2	0.83 (0.81-0.85)	168.7	0.86 (0.84-0.89)	151.2	0.96 (0.92-1.00)
2	103	20.7	121.4	0.85 (0.81-0.89)	114.0	0.90 (0.86-0.96)	92.3	1.12 (1.03-1.21)
3+	88	17.7	78.6	1.13 (0.98-1.29)	82.8	1.07 (0.93-1.22)	90.4	0.98 (0.87-1.11)

O = Observed, E = Expected, CI = Credible Interval.

^1^Controlling for age, specific HPV prevalence.

^2^Controlling for age, specific HPV prevalence, CD4 counts, cART and interaction term between CD4 and cART.

^3^As^2^ plus individual random effects.

3+ means 3 HPV types or more.

**Figure 1:**
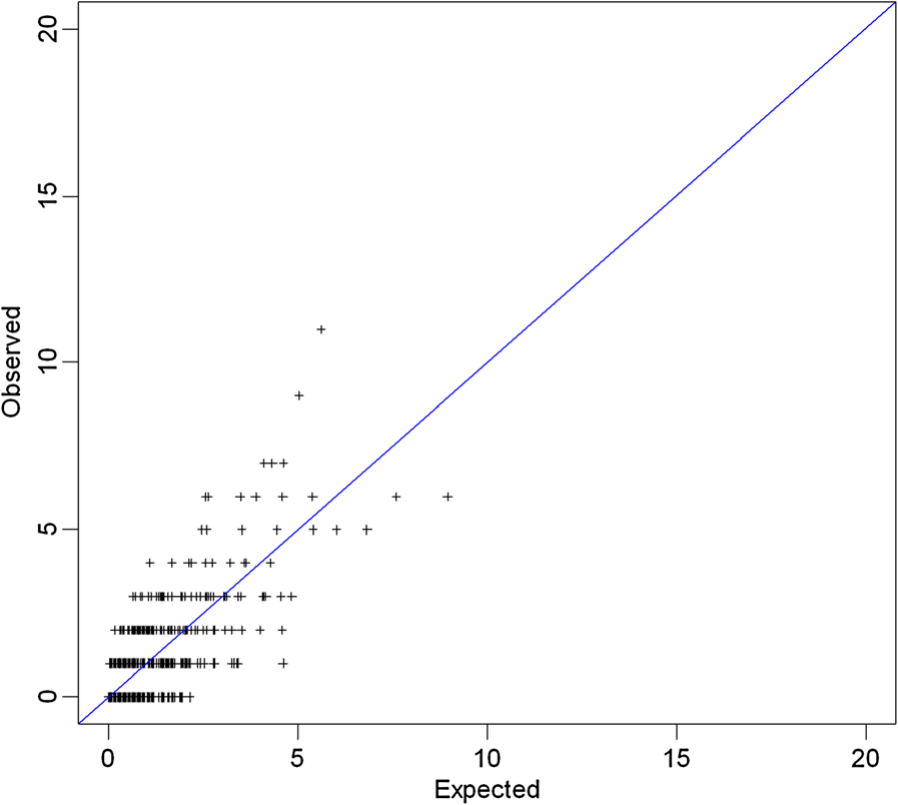
**Observed versus Expected occurrence for 2**-**way combinations of 44 HPV types**, **HIV**-**positive women in Kenya.** Plus signs represent occurrences of HPV pairs. HPV pairs located in the upper triangle indicate positive clustering, while those located in the lower triangle represent negative clustering between the HPV types involved. None of the p-values for joint HPV infections was significant at the chosen significance level of 0.00005. No significant associations emerged even when considering a less conservative level, p < 0.01.

No significant deviations of O/E counts were observed for each 2-way combination of 44 HPV types (Figure 1). None of the pairs of HPV types reached the chosen level of significance, p = 0.00005. No significant excess or deficit of 2-way combinations of types was found when we used a less conservative level of significance (p = 0.01) (data not shown). The results of the analyses were substantially similar when women with CIN2/3 were excluded from the analyses and when stratified by cART use (never; ever) and CD4 count (< or ≥350 cells/μL) (data not shown).

## Discussion

The present study of HIV-positive women in Kenya found that there was no evidence of either an excess or a lack of clusters between specific HPV types, even though a slight excess of infections with 2 HPV types was observed even after controlling for all sources of common correlation between HPV types (random effects).

Our study is the first to evaluate clustering patterns of specific HPV types among HIV-positive women in Africa. The assessment of the associations between all the different HPV genotypes requires studies with a high statistical power. The present study was relatively small compared to previous studies in the general population [3,5,6,8]. Small size was, however, partly compensated by the larger number of multiple HPV infections in HIV-positive women.

A strength of our analysis was the inclusion into the logistic regression model of random effects at the individual level that allowed us to account for the unobservable risk factors shared by all HPV types. Non type-specific clustering of HPV types has often been observed in previous studies of HIV-negative women prior to the inclusion of random effects. Non type-specific clustering has generally been explained by a correlation with high-risk sexual behavior as all HPV types are sexually transmitted [3,5,6,8]. In our study of HIV-positive women from Kenya, however, multiple HPV infections were associated with the degree of immunosuppression and not with sexual behavior [10]. Immunosuppression diminishes the probability of HPV clearance and increases the risk of HPV reactivation [16]. Random effects, however, contributed substantially to reducing the spurious discrepancy between observed and expected numbers of infections, with the possible exception of double infections.

In conclusion, our present findings confirm in HIV-positive women previous results observed in HIV-negative women, providing further evidence on the lack of clustering between carcinogenic HPV types in either immunocompetent or immunosuppressed women.

## Abbreviations

cART: combination antiretroviral therapy; CI: Credible Interval; HPV: Human papillomavirus; O/E: Observed-to-expected.

## Competing interests

The authors declare that they have no competing interests.

## Authors’ contributions

MHC, HDV, NRM and SF conceived and designed the study. SV performed the statistical analyses. MHC, NRM, and SRS contributed to data collection. DAMH performed the HPV testing. All authors contributed to the interpretation of the findings, and SV, HDV and MHC wrote the manuscript. All authors approved the final manuscript.

## References

[B1] Clifford GM Goncalves MA Franceschi S, for the HPV and HIV Study Group: human papillomavirus types among women infected with HIV: a meta-analysis AIDS 2006 8 2337 2344 10.1097/01.aids.0000253361.63578.14 17117020

[B2] De Vuyst H Lillo F Broutet N Smith JS HIV, human papillomavirus, and cervical neoplasia and cancer in the era of highly active antiretroviral therapy Eur J Cancer Prev 2008 8 545 554 10.1097/CEJ.0b013e3282f75ea1 18941376

[B3] Vaccarella S Franceschi S Snijders PJ Herrero R Meijer CJ Plummer M Concurrent infection with multiple human papillomavirus types: pooled analysis of the IARC HPV Prevalence Surveys Cancer Epidemiol Biomarkers Prev 2010 8 503 510 10.1158/1055-9965.EPI-09-0983 20142247

[B4] Chaturvedi A Katki H Hildesheim A Rodriguez AC Quint W Schiffman M Van Doorn LJ Porras C Wacholder S Gonzalez P Sherman M Herrero R Human papillomavirus infection with multiple types: pattern of co-infection and risk of cervical disease J Infect Dis 2011 8 910 920 10.1093/infdis/jiq139 21402543 PMC3068034

[B5] Vaccarella S Franceschi S Herrero R Schiffman M Rodriguez AC Hildesheim A Burk RD Plummer M Clustering of multiple human papillomavirus infections in women from a population-based study in Guanacaste, Costa Rica J Infect Dis 2011 8 385 390 10.1093/infdis/jir286 21742837 PMC3132145

[B6] Carozzi F Ronco G Gillio-Tos A De Marco L Del Mistro A Girlando S Franceschi S Plummer M Vaccarella S Concurrent infections with multiple human papillomavirus (HPV) types in the New Technologies for Cervical Cancer (NTCC) screening study Eur J Cancer 2012 8 1633 1637 10.1016/j.ejca.2011.10.010 22088483

[B7] Vaccarella S Soderlund-Strand A Franceschi S Plummer M Dillner J Patterns of human papillomavirus types in multiple infections: an analysis in women and men of the high throughput human papillomavirus monitoring study Plos One 2013 8 e71617 10.1371/journal.pone.0071617 23977090 PMC3747214

[B8] Vaccarella S Plummer M Franceschi S Gravitt P Papenfuss M Smith D Villa L Ponce EL Giuliano AR Clustering of human papillomavirus (HPV) types in the male genital tract: the HPV in men (HIM) study J Infect Dis 2011 8 1500 1504 10.1093/infdis/jir595 21908729 PMC3222106

[B9] Chaturvedi AK Myers L Hammons AF Clark RA Dunlap K Kissinger PJ Hagensee ME Prevalence and clustering patterns of human papillomavirus genotypes in multiple infections Cancer Epidemiol Biomarkers Prev 2005 8 2439 2445 10.1158/1055-9965.EPI-05-0465 16214929

[B10] De Vuyst H Mugo NR Chung MH McKenzie KP Nyongesa-Malava E Tenet V Njoroge JW Sakr SR Meijer CM Snijders PJ Rana FS Franceschi S Prevalence and determinants of human papillomavirus infection and cervical lesions in HIV-positive women in Kenya Br J Cancer 2012 8 1624 1630 10.1038/bjc.2012.441 23033006 PMC3493776

[B11] Chung MH McKenzie KP Richardson BA John-Stewart GC Coombs RW De Vuyst H Njoroge JW Nyongesa-Malava E Sakr SR Mugo NR Cervical HIV-1 RNA shedding after cryotherapy among HIV-positive women with cervical intraepithelial neoplasia stage 2 or 3 AIDS 2011 8 1915 1919 10.1097/QAD.0b013e32834a3654 21716072 PMC3248579

[B12] Chung MH McKenzie KP De Vuyst H Richardson BA Rana FS Pamnani R Njoroge JW Nyongesa-Malava E Sakr SR John-Stewart GC Mugo NR Comparing pap smear, via, and hpv cervical cancer screening methods among hiv-positive women by immune status, and antiretroviral therapy AIDS 2013 [Epub ahead of print] 10.1097/01.aids.0000432472.92120.1b PMC4007364 23842133

[B13] van den Brule AJ Pol R Fransen-Daalmeijer N Schouls LM Meijer CJ Snijders PJ GP5+/6+ PCR followed by reverse line blot analysis enables rapid and high-throughput identification of human papillomavirus genotypes J Clin Microbiol 2002 8 779 787 10.1128/JCM.40.3.779-787.2002 11880393 PMC120256

[B14] Plummer M Vaccarella S Franceschi S Multiple human papillomavirus infections: the exception or the rule? J Infect Dis 2011 8 891 893 10.1093/infdis/jiq146 21402540

[B15] Vaccarella S Plummer M Franceschi S Reply to Cervantes J Infect Dis 2011 8 1816 181721987666

[B16] Strickler HD Palefsky JM Shah KV Anastos K Klein RS Minkoff H Duerr A Massad LS Celentano DD Hall C Fazzari M Cu-Uvin S Bacon M Schuman P Levine AM Durante AJ Gange S Melnick S Burk RD Human papillomavirus type 16 and immune status in human immunodeficiency virus-seropositive women J Natl Cancer Inst 2003 8 1062 1071 10.1093/jnci/95.14.1062 12865452

